# Moroccan Strawberry Tree (*Arbutus unedo* L.) Fruits: Nutritional Value and Mineral Composition

**DOI:** 10.3390/foods10102263

**Published:** 2021-09-24

**Authors:** Zakaria Ait lhaj, Rahma Bchitou, Fatima Gaboun, Rabha Abdelwahd, Tarik Benabdelouahab, Mohammed Rachid Kabbour, Paul Pare, Ghizlane Diria, Khadija Bakhy

**Affiliations:** 1 Research Unit on Plant Breeding and Conservation of Plant Genetic Resources and Biotechnology, National Institute of Agricultural Research (INRA), Rabat BP 6570, Morocco; aitlhajzakaria.smc@gmail.com (Z.A.l.); fatima.gaboun@inra.ma (F.G.); rabha.abdelwahd@inra.ma (R.A.); tarik.benabdelouahab@inra.ma (T.B.); mohammedrachid.kabbour@inra.ma (M.R.K.); ghizlane.diria@inra.ma (G.D.); 2 Center of Materials Science, Nanostructures Laboratory, Process Engineering and Environment, Faculty of Sciences, Université Mohammed V de Rabat Siège, Rabat 10000, Morocco; bchitou@hotmail.com; 3 Department of Chemistry & Biochemistry, Texas Tech University, Lubbock, TX 79409, USA; paul.pare@ttu.edu

**Keywords:** *Arbutus unedo* L., paraphyletic variability, wild edible fruit, nutritional value, mineral composition

## Abstract

The strawberry tree (*Arbutus unedo* L.), grown throughout the Mediterranean, produces edible fruit; as it is easily bruised, the sweet, reddish fruit is used mostly to prepare jams, marmalades and alcoholic beverages. As the genus is paraphyletic, phytochemical analysis can assist in defining the fruit composition with the species *Arbutus unedo* L. (*A. unedo*). Here we report on the carbohydrate, total sugar, protein, fat, fiber, ash, and mineral content of wild fruit, harvested from 45 specimens from five locations. The dominant nutrients were carbohydrates (78.2–84.8 g/100 g), total sugars (52.1–67.2 g/100 g) and dietary fiber (11.0–20.1 g/100 g). Other important nutrients supplied by *A. unedo* fruit include P, K, and Fe. The fruit was observed to contain health-promoting components providing 42 and 36%, of recommended daily allowance (RDA) for fiber and zinc, respectively, as well as iron and manganese, at levels exceeding minimum RDA. The free-sugar profile revealed high glucose followed by fructose content with minor amounts of sucrose (14, 11, and 6 g/100 g, respectively). Significant differences both between regions and within individuals were observed for several traits. The richness of fruit nutrients in *A. unedo* confers nutritional value and as such, a promising alternative fruit source.

## 1. Introduction

*Arbutus unedo* belongs to the Ericaceae family and produces an edible red fruit (ca. 2 cm in diameter) [1]. The shrubby ornamental tree matures to ca. 3 m in height and in the winter blooms with pinkish-white flowers. The fleshy fruit is often consumed as a jam or used in the manufacture of digestive and/or laxative liqueurs [2,3,4,5]. Recent reports point to a role of *A. unedo* extracts in treating type-2 diabetes, by inhibiting α-amylase activity. Plant extracts also exhibit therapeutic potential against Alzheimer’s and Parkinson’s diseases due to the presence of arbutin an inhibitor that blocks disease progression [4]. Moreover, the consumption of such wild fruit can enhance nutritional uptake as well as natural antioxidant levels [6].

Specifically, concerning the fruit, the most abundant chemical component is carbohydrates that present almost 40% of the total weight of fresh berries, showing an average value of 938.3 ± 4.1 gkg^−1^ DW [7]. According to Barros et al. [7], the composition of carbohydrates in fruits consists mainly of monosaccharides (fructose and glucose), disaccharides (sucrose), and polysaccharides (cellulose and starch). Furthermore, it has been reported that their levels vary considerably, depending on the ripeness stage of the fruit. In this view, sucrose is the most abundant saccharide in the unripe fruits (208 ± 2 g kg^−1^ DW), while fructose is the major free sugar in the ripe stage, reaching an average value of 88 ± 0.6 g kg^−1^ DW [8]. Besides the saccharides, organic acids are also affected by the maturation stages of the fruit [9]. Indeed, α-linolenic acid is the most abundant fatty acid in different stages of ripening, followed by oleic acid and linoleic acid as the less abundant compound (with relative percentages ranging from 37 ± 1.8 to 43 ± 0.2, 29 ± 1.8 to 27 ± 0.2 and 20 ± 0.6 to 18.8 ± 0.1 in unripe and ripe fruits, respectively). Overall, fatty acid fruit composition includes almost 60% polyunsaturated fatty acids (PUFA), providing a high favorable ω-3/ω-6 ratio due to the potent rate of α-linolenic acid [9]. Therefore, it has been reported that the tasty flavor characterized the fruits in their advanced stage of maturity, and sensory characteristics are principally due to the combination of sugar and fatty acids, which makes the berries subject to traditionally uses in the production of alcoholic beverages [4]. Moreover, this fruit is a great source of vitamin C and dietary fiber; it also contains high levels of the vitamin E component α-tocopherol, which contains potent antioxidant activity [4].

The berries as an abundant source of biologically active compounds, their fatty acid content includes ω-3 PUFA, properly supplemented with potent vitamin E rates allowing them further utilization as dietary supplements or functional food [4,9,10]. At the international level, several studies showed the beneficial potential of *A. unedo* fruit as well as a bioactive phytochemical source in addition to their nutritional aspect [4,11,12].

Despite the benefits of fruits, their use at an industrial scale and their plantation have not yet been achieved, because there is a lack of screening in their growing area to identify varieties for higher production and best quality of fruits [13]. Indeed, notwithstanding that the plant is grown throughout the Mediterranean region that includes Southern Europe and North Africa [13,14], variability in fruit chemical composition has not been investigated [15]. The nutraceutical value of *A. unedo* fruit was based on single-site investigations [8,16,17,18]. In Morocco, a few studies revealed the chemical profile and biological activities of strawberry-tree fruit, where it has been proved an antioxidant activity of his aqueous extract [5,19]. Therefore, to achieve representative data on the composition of this fruit, widely distributed in Morocco, we aim to assess the chemical composition of *A. unedo* fruits gathered from different Moroccan regions. The goal is to check whether the fruit can be used locally in the food-processing industry to enhance the plant’s economic value. For this purpose, chemical analysis was performed, including moisture, pH, proteins, fats, dietary fiber, total sugar, mineral composition, ash, and energetic value; individual sugar profiles were also examined via chromatographic technique.

## 2. Materials and Methods

### 2.1. Plant Material and Experimental Areas

**Figure 1:**
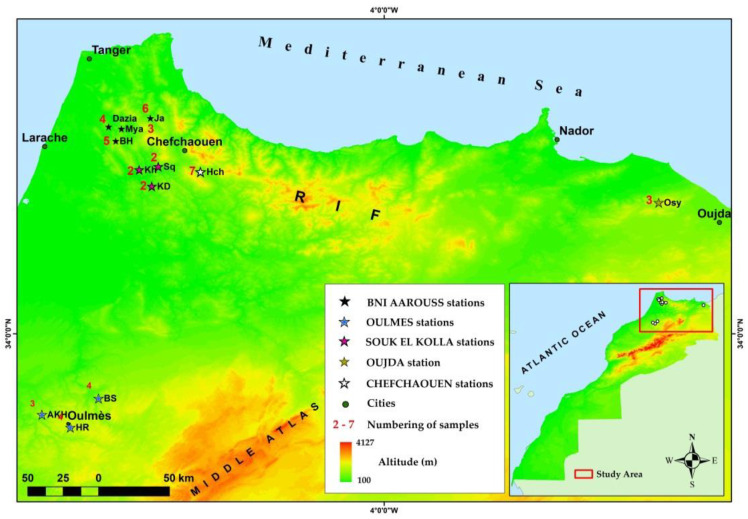
Sampling points and geographic origin of the *A. unedo* surveyed populations.

*A. unedo* L berries of 2–7 individual trees were collected from 12 stations covering five Moroccan areas, (Figure 1). Data about samples number and altitude of the sampling stations are depicted in the (Table 1). In total, 45 samples were collected. The harvest was carried out in November 2017 when fruits reach their optimal maturity status. The ecological and geographical characteristics of each station were noted. Each sample had at least 1kg of uniform ripeness fruits. All samples were put in tagged plastic bags inside an isothermal container and transported in the laboratory of the National Institute of Agriculture Research (INRA). All samples were lyophilized and kept in the best conditions for ensuring use. The Specimens of the investigated plant were taxonomically identified by Pr. Hamid Khamar at the National Scientific Institute of Rabat (Department of Botany and Plant Ecology). A voucher specimen was deposited in the National Herbarium of Scientific Institute of Rabat and registered under the number code RAB111500.

### 2.2. Analytical Methods

Aliquots of each sample were taken to analyze dry matter and pH. Dry matter (DM) was determined using an oven at 100 ± 3 °C until desiccation to constant weight according to the AOAC method, and homogenized sample 1/10 (*w/v*) in distilled water was used to measure pH by potentiometer [20]; triplicate subsamples were taken for all analytical procedures.

#### 2.2.1. Nutrients Composition

The nutritional composition of fruit samples was evaluated according to the Association of Official Analytical Chemists (AOAC) procedures. The ash content was determined by incineration of 2 g of fruit sample in a muffle furnace for six hours at 550 °C [20]. Fat content was determined by extracting 5 g of lyophilized fruits with petroleum ether using the Soxhlet apparatus, [21]. Protein content (PC) was estimated by the macro-Kjeldahl method, by using the conversion factor 6.25 to converting total nitrogen content (N) quantified through the digestion of fruit powder with concentrated sulfuric acid to protein content [22].

The amount of crude fiber (CF) was estimated following the AOAC method [22], through the application of sulfuric acid and sodium hydroxide to hydrolyze the non-cellulosic fraction in powder of fruit samples. Total carbohydrates were calculated by applying the formula: Total carbohydrates (TC) = 100 − (moisture + g protein + g fat + g ash) and the energetic value is expressed by the equation:

Energy (kcal) = 4 × (g protein + g carbohydrate) + 9 × (g lipid)(1)

Total sugar contents (TSC) were determined by spectrophotometric method using phenol-sulfuric acid reagent [23], the absorbance was measured at 490 nm, using a UV/Vis Spectrometer (Model UV2601 UV/VIS/Rayleigh, Beijing Rayleigh Analytical Instrument Corporation (BRAIC/China). Briefly, 1ml of fruit extracts (ethanol/water solution (80/20 *v/v*)) was mixed with 1 mL of phenol reagent (5%) and 5 mL of sulfuric acid, after a vigorous agitation of the mixture, it kept in the water bath at 40 °C for 30 min. The total sugar contents were quantified against a standard glucose calibration curve (concentration range of 0.004 mg/g to 1 mg/g).

#### 2.2.2. Soluble Sugars

The sugar composition was determined for a composite sample of each region. The composite sample was a mixture of all the individuals of each region. So, the soluble sugar profile was analyzed for only 5 samples corresponding to 5 regions.

**Figure 2:**
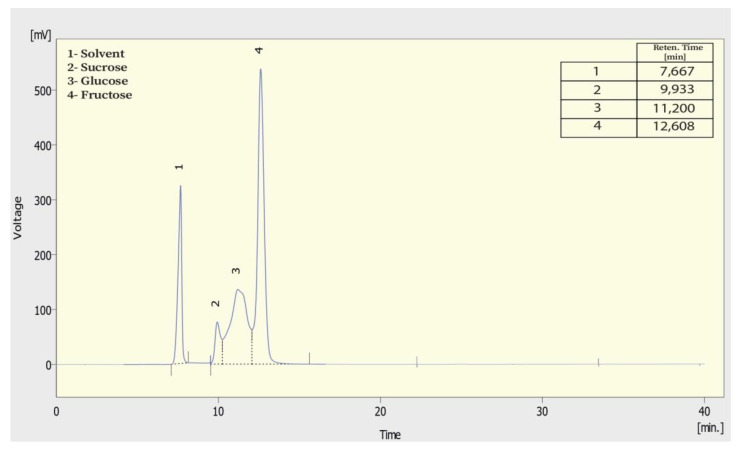
HPLC chromatogram of standards sugar (concentrations of fructose, glucose, and sucrose are 1.957, 3317, and 1.851 g/100 mL, respectively).

The identification of soluble sugars of *A. unedo* fruit was made by high-performance liquid chromatography coupled to a refraction index detector (HPLC-RI) according to the procedure described by Barros et al. [7]. Freeze-dried sample powder (1.0 g) was extracted with 40mL of aqueous ethanol, 20:80 (*v:v*). The mixture was centrifuged for 10 min, and then it was filtered, and concentrated at 60 °C under reduced pressure, and degreased threefold with 10 mL of ethyl ether. After submitting a heat treatment at 40 °C to concentrate the extracts, the residual part was dissolved in distilled water (5 mL). Free sugars contents were detected by using HPLC system equipped with YL9170 Refractive Index Detector at 35 °C and with SUPELCOGEL^™^ C610H, 6% Crosslinked HPLC Column (9 µm, 30 × 0.78 cm) and a temperature column of 50 °C. The mobile phase was 0.1% of ortho-phosphoric acid (H_3_PO_4_) in deionized water, at a flow rate of 0.8 mL/min. All obtained chromatograms (Figure 2) were dealing with YL-Clarity data system; the identification of free sugar was realized by comparing the relative retention times of sample peaks with standards and the content in each compound was calculated against calibration curves acquired by using external standard method (concentration ranges of 0.41–3.31 g/100 mL, 0.24–1.95 g/100 mL and 0.23–1.85 g/100 mL; in glucose, fructose and sucrose, respectively). All results are expressed in g/100 g of dry weight.

#### 2.2.3. Mineral Composition

The ash content was determined according to the AACC method [22], by incineration of 2 g of sample in a muffle furnace for 6 h at 500 °C. The residue is then cooled in a desiccator and weighed. For the mineral composition, 1 g of the residue freeze-dried samples was incinerated in a muffle furnace for 6 h at 500 °C and extracted with saturated HCl, the ashes portion is recuperated in a jogged flask and made up to an appropriate volume (100 mL) with distilled water. The mineral contents of Fe, Mn and Zn were measured by atomic absorption spectrophotometry in flame air-acetylene. Potassium (K) and Sodium (Na)were determined by using a flame photometer, phosphorus (P) content was estimated by the reaction of the acidified solution of ammonium molybdate, ascorbic acid, and antimony [24]. The results were determined at optical density 825 nm with a UV-visible spectrophotometer (Model 6405 UV/VIS spectrometer/Jenway/UK).

#### 2.2.4. Statistical Analysis

All the assays were carried out in triplicate, including extraction processes. The results are expressed as mean ± SE (Standard Error). The data were analyzed by using analysis of variance (ANOVA) followed by Duncan’s Multiple Range Test at 95% confidence level to determine the significant differences among the means of studied compounds. A matrix comprising nutritional compounds values for each sample and harvest location was subjected to a principal compound analysis (PCA) and Discriminant Factorial Analysis (DFA) to summarize the principal factors determining the differences of these parameters and to classify the samples into a principal group. These statistical analyses were conducted by using SAS (version 9.1) and GenStat software (18th edition) successively.

## 3. Results

### 3.1. Nutritional Composition

Individual trees of *A. unedo* fruits have been collected from the Moroccan sites where they grow spontaneously. In detail, 45 samples have been collected in 12 stations covering most of the natural habitats of the species in Morocco (Figure 1). The average nutritional composition and energetic value (expressed on a dry weight basis (DW)) of the 45 samples of *A. unedo* fruits are presented in (Table 2). The most abundant macronutrient was carbohydrates (81.59 ± 0.12 g/100 g), total sugar (60.95 ± 0.28 g/100 g), dietary fiber (16.07 ± 0.17 g/100 g) and moisture (9.87 ± 0.10 g/100 g). However, fat, Protein, and ash were found to be less abundant macronutrients in Moroccan berries with contents of 3.44 ± 0.06, 2.83 ± 0.04, and 2.24 ± 0.03 g/100 g, respectively. All samples show an acid character with an average value of 3.9. The highest variation among the individuals surveyed was observed for fat, Ash, protein, dietary fiber, and moisture.

Table 3 summarizes the average content of mineral components of *A. unedo* fruits. The strawberry tree fruits’ mineral profile was characterized by the abundance of P, K, Fe, and Na. Phosphorus (P), potassium (K), and iron (Fe) showed high content with mean values of 63.7 mg/100 g DW, 57.4 mg/100 g DW, and 28.7 mg/100 g DW, respectively, while sodium (Na) was found at a content reaching 15.1 mg/100 g. Mn and Zn were detected at low concentrations (3.9 mg/100 g). Na showed a wider variability range (1–83 mg/100 g). Similarly, for all the microelements, the range of variation was very high. P and K were quite variable, among the individuals, ranging between 37.6 and 96.6, and 39 and 89 mg/100 g.

### 3.2. Nutritional Value Variability

**Figure 3:**
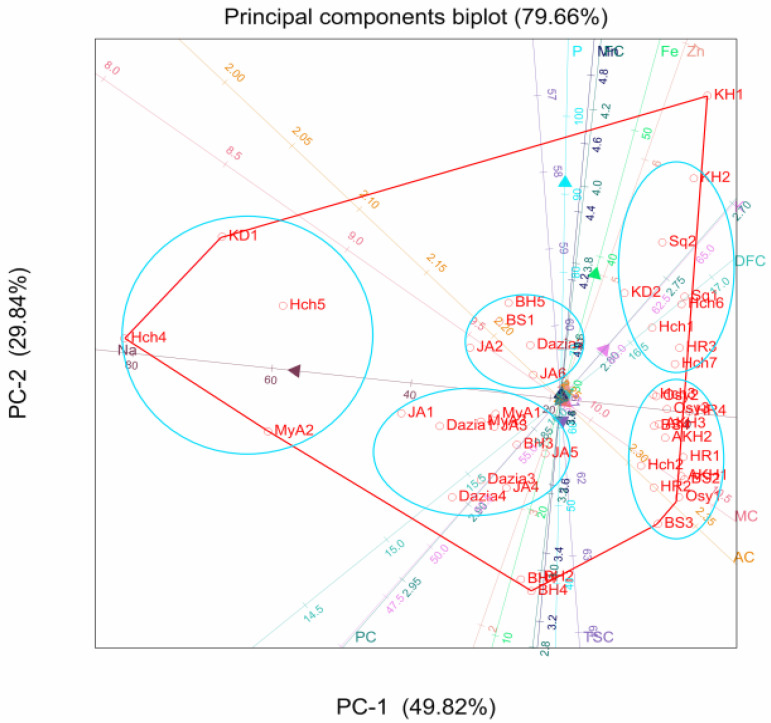
The PCA of the forty-five surveyed samples using the first two principal component axes. BA (BH1-BH5, DZ1-DZ4, JA1-JA6, MYA1-MYA3); CH (Hch1-Hch7); OUJ (Osy1-Osy3); OUL (AKH1-AKH3, BS1-BS4, HR1-HR4) and SQ (KD1-KD2, Kh1-Kh2, Sq1-Sq2).

The 45 nutritional compositions were subjected to principal component analysis (PCA) which was realized based on the 12 traits (Moisture, protein, pH, dietary fiber, fat, total sugar, ash, P, K, Na, Fe, Zn, Mn). The two first principal components (PC1) and (PC2) explained 79.7% of the total data variance (Figure 3). The first principal component (PC1 = 49.8%) displayed a strong negative correlation with sodium content. PC1 separated *A. unedo* plants rich in sodium from those that contain intermediate or low amounts. On the other hand, PC2 was positively and strongly related to phosphorus and iron content.

Regarding PC1, four from the 45 samples (Hch4, Hch5, MyA2, and KD1) have the strongest negative scores, indicating that they are characterized by high amounts of Na. Most samples from OUL (82%), all from OUJ, one from BA (MyA3), and CH(Hch2), showed positive scores on account of their lowest amount of Na. The remaining samples which grouped most samples of BA and OUL were characterized by intermediate content of Na. Concerning PC2, nine samples of which mostly are from SQ (5 samples), three from CH (Hch1, Hch6, and Hch7), and one from OUL showed intermediate to high positive scores, implying their high phosphorus content. All samples from the BA region except MyA2 and one from OUL (BS1) showed intermediate to high negative scores, implying their intermediate to low phosphorus content, even if, some samples have particularly low positive scores.

**Figure 4:**
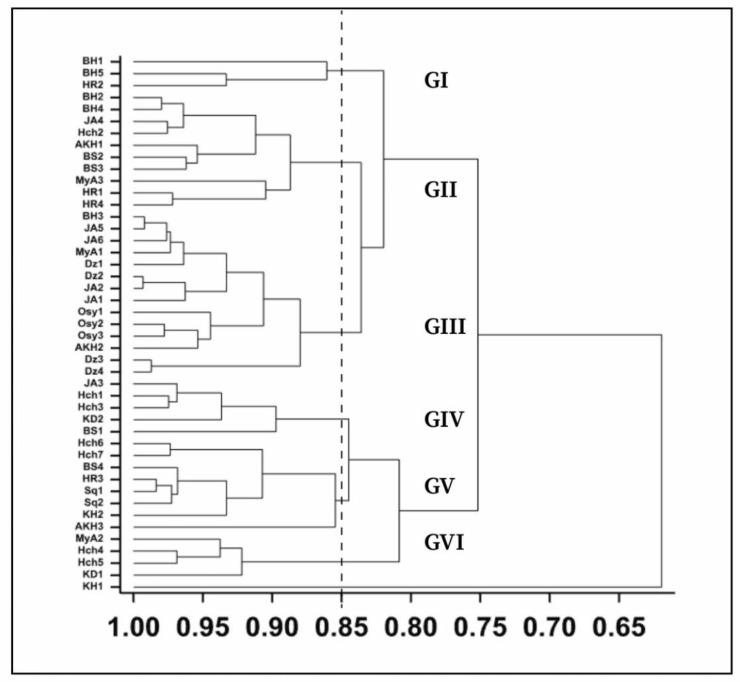
Hierarchical clustering Dendrogram showing the relationships among the forty-five samples belong to the surveyed regions.

Cluster Analysis (CA) was performed to classify and differentiate the analyzed *A. unedo* samples according to their major constituents. Figure 4 presents the corresponding dendrogram using Ward’s method [25] Considering the 12 major constituents of the 45 samples, six major groups and one ungrouped sample were defined whose composition is summarized in (Table 4).

The resulting dendogram reported in (Figure 4) reflects the qualitative heterogeneity of wild Moroccan *A. unedo* and showed the existence of high intrapopulation variability within the samples. The following groups have been defined:

Group I: This group gathers three samples, two from BA and the third from OUL, and is characterized by noticeable amounts of P (mean = 58.9 ± 16.6 g/100 g DW), K (mean = 57.9 ± 9.5 g/100 g DW), Fe (mean = 19.53 ± 15.0 g/100 g DW) and Na (mean = 17.2 ± 12.4 g/100 g DW) (Table 4).

Group II: represents 22.2% of samples and shows higher content of Fe (mean of 28.3 ± 13.5 g/100 g DW) while lower sodium content than Group I, (10.6 ± 11 g/100 g DW) (Table 4). The samples consist of 45% of OUL samples and 22% of BA and one from CH (Hch2).

Group III: This group is the largest in terms of the number of samples including 14 individuals (31% of samples) and represents a profile rich in Fe (mean of 27.9 ± 8.5 g/100 g DW). Phosphorus (59.6 ± 8.42 g/100 g DW), potassium (55.5 ± 4.2 g/100 g DW), and sodium (21.4 ± 13.1 g/100 g DW) were the most relevant components (Table 4). This group gathers all samples of the OUJ region, 56% of BA, and one come from OUL (AKH2).

Group IV: This group consists of five samples BA (1 sample), OUL (1 sample), CH (2 samples), SQ (1 sample). It was characterized by the preeminence of phosphorus (72.4 ± 6.52 g/100 g DW), potassium (59.3 ± 4.3 g/100 g DW), iron (27.7 ± 6.8 g/100 g DW), and appreciable content of Na (15.2 ± 11.6 g/100 g DW) (Table 4).

Group V: Among this group, three samples are localized in OUL, three in SQ, and two samples in CH. As well as group IV, this group reached high content of phosphorus (77.4 ± 8.7 g/100 g DW), potassium (63.5 ± 11.5 g/100 g DW), and iron (30 ± 12.4 g/100 g DW). This group presented the lowest Na content (3.17 ± 1.4 g/100 g DW) (Table 4).

Group VI: this group is considered sodium-rich (68 ± 9.8 g/100 g DW). It is the least frequent one and consists of 4 individuals sampled from CH (2 samples), SQ, and BA (1 sample for each). This group is also characterized by high amounts of phosphorus (70.4 ± 12.8 g/100 g DW), potassium (53.5 ± 4.4 g/100 g DW), and iron (29.3 ± 3.3 g/100 g DW).

Ungrouped sample: (KH1) from the SQ region, was the most dissimilar individual that presented the richest mineral composition with high content in all surveyed mineral elements (P, K, Fe, Zn, and Mn) except for sodium, where it exhibits the lowest amount with a mean value of 3.33 g/100 g DW (Table 4).

### 3.3. Geographical Repartition of Mineral Composition

**Figure 5:**
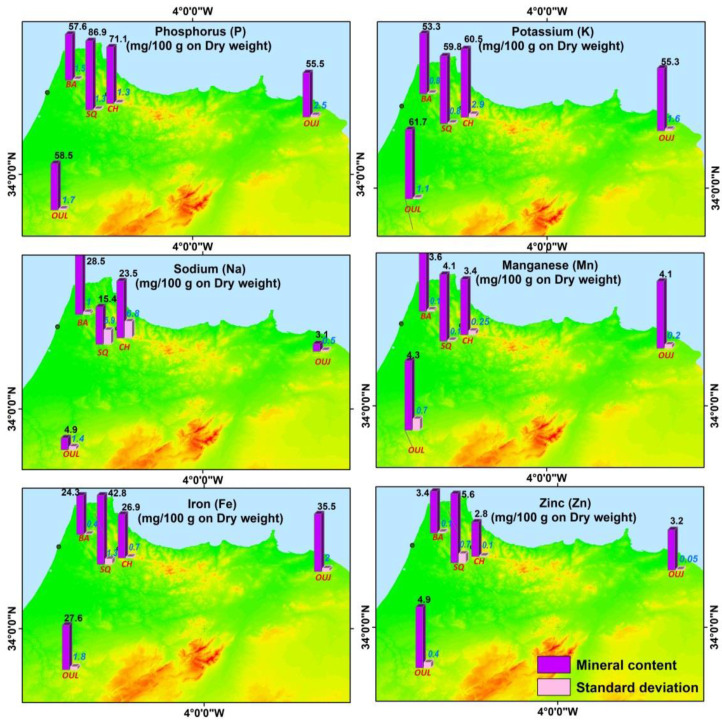
Geographical distribution of average mineral content and standard deviation on the surveyed area.

When considering the geographical distribution of mineral composition. Total ashes content was highly stable in the region; however, the behavior of the different mineral elements studied was quite different. Origin of the fruit had consistent and significant effects on all mineral variables except Mn. The SQ had more phosphorus, iron, and zinc. Potassium and manganese concentrations were greater for the OUL fruit but contained less phosphorus and iron. On the other hand, Na was less abundant in OUL and OUJ regions (Figure 5).

### 3.4. Soluble Sugars Profile

The sugar composition (Table 5) was characterized by the abundance of glucose and fructose in all samples over surveyed regions with a range of 11.6 to 15.2 g/100 g and 8.7 to 13.1 g/100 g, respectively, while sucrose was showed in the lowest levels (content ranged between 4.2 and 8.1 g/100 g). When comparing the regions, fruit from the BA contained the highest glucose and fructose. OUL and OUJ showed similar content and are the second richest region according to the content of glucose and fructose. The greatest content of sucrose was recorded in CH and OUJ fruit while the lowest contents in OUL, BA, and SQ.

## 4. Discussion

In the present study, data on the nutritive and mineral composition of Moroccan *A. unedo* fruit were obtained. The nutrient content, including total protein, fat, ash, carbohydrates, sugar profile, and minerals was evaluated in fruits of almost strawberry tree populations range in Morocco. pH values presented significant variability (*p* < 0.001) among the different regions. It ranged between 3.78 and 4.19 and was similar to those found in some Mediterranean areas: The Algerian fruit (with an average value of 3.43) in the study reported by Boussalah et al. [26], and 3.2 to 3.5 in Spain which has proven no significant variation within year and harvest areas [10]. This parameter has an essential advantage at the industrial level, especially for juice, jelly, and jam production. Moisture in lyophilized fruit was within the range of 9.4% to 10.9%, these results showed an average value higher than the main content (7.5%) reported by Nunes [4]. Here the mode of lyophilization was used to dehydrate the fruits by preventing any degradation of the chemical composition that can take place by thermal drying. It was also reported that the moisture contents of fresh fruits were within the range of 43.7% to 67.9%, depending on the harvest locations [4].

Carbohydrates calculated by subtracting the other main nutrients (moisture, protein, fat, and ash) from the total, were the most abundant compound and higher than 80% but less than the content found in Portugal (an average value of 94.02%) [4]. Furthermore, a small variation range was showed between fruits samples collected from the different regions (with 81% to 83%, in OU and CH regions, respectively). The important content of this macronutrient in fruits could be explained by their need for a considerable quantity of carbohydrates for seed germination [4], also it was reported that strawberry fruits are rich in several carbohydrates mainly starch and cellulose [16].

Another macronutrient found in Moroccan fruit is dietary fiber, with an average value (16%) higher than the content proved in Algerian strawberry-tree fruits (average value over 6%), this suggests that fruits gathered from Moroccan areas are an important dietary source for fiber with respect to plants grown in Algeria [26]. This establishes the importance of the North African *A. unedo* fruit, especially those gathered from Morocco, as a dietary alternative to enrich modern diets which are often devoid of dietary fiber.

As depicted in (Table 6), 100 g of fruits could provide 42% of the daily recommendation of fiber for men and 64% of the daily portion required for women (the recommendation of fiber intakes are 38 g/day for males and 25 g/day for females) [27]. These findings are confirmed by Ruiz-Rodríguez et al. [10], who found almost similar daily amounts required (42.6 versus 64.8% for men and women, respectively). However, significant variation was observed over surveyed regions in this nutrient with range values of 15 to 19%.

For crude fat, the minor component of the fruit varied from 3.3 to 3.8% and showed significant variability among regions but without a significant influence of water content. Those results were higher than those found in all previous studies, which reported contents of 0.7, 1.4%, and 0.6% in Algerian, Portuguese and Spanish fruit, respectively [7,10,26].

Protein was found to be a less abundant macronutrient in Moroccan berries, with a content ranging from 2.3 to 3.5 g/100 g. These results were consistent with those obtained in Algeria (2.3–3.6 g/100 g) [26], but with an average value (2.8 g/100 g) higher than that reported by Ruiz-Rodríguez et al. [10], which showed an average protein content of 0.9 g/100 g. According to recommended dietary allowances (RDA) referred by Trumbo et al. [27], which recommend a protein intake of 56 g/day and 46 g/day for men and women, respectively, 100 g of fruits can provide 5.1% and 5.2% of the daily serving required for men and women, respectively. By comparing these results to RDA in Spanish fruit (1.6% f and 1.9% of RDA for men and women, respectively) reported by Ruiz-Rodríguez et al. [10], it can be demonstrated that Moroccan fruits can be considered the highest contributor to protein intake.

Based on the contents of the most important macronutrients in *A. unedo* fruit, it can be assessed that a dry portion assures energetic values between 365 and 372 Kcal/100 g (DW) of fruit, with an average value coincidental with the energetic value (400 Kcal/100 g) reported by Barros et al. [7], and it was raised more than three times the energetic value (101 Kcal/100 g) found in Algerian fruit [26]. The variance in this parameter is attributed to carbohydrates content; this is proved by the positive correlation observed between the carbohydrates contents in samples over surveyed regions and their energy contribution, except the samples collected from OUJ and BA since they had significantly different fiber content and close energetic values (18.70 and 14.96% of dietary fiber; 80.85 and 81.73% of carbohydrates, in OUJ and BA, respectively).

Moroccan berries revealed the highest sucrose content (average value of 6.2 g/100 g) in regards to the values found in Portuguese, Spanish and Turkish fruits (average values of 4.2, 0.4, and 1.8 g/100 g, respectively) [7,10,28], while it was absent in all samples collected from different Algerian sites [26]. Those differences could be explained by the enzymatic hydrolysis of sucrose into monosaccharides (glucose and fructose), especially during the fruit ripening process [28]. The composition of free sugars proved in this study is the first report of glucose as the most abundant saccharide, followed by fructose, while most previous studies have shown a high contribution of fructose followed by glucose [7,10,26,28,29].

Hence, the ratio glucose/fructose was in the range of 1.2 to 1.3, with higher fluctuations comparing with glucose/fructose ratios reported by Ruizíguez et al. [10], and Boussalah et al. [26] (ratio values ranged from 0.4 to 0.6 and 0.55 to 0.65, respectively). It was reported that the fruit which provides a ratio of glucose/fructose near 1, has a high invertase activity (that means the ability to convert sucrose in glucose and fructose) such as kaki and prickly fruits [28]. This finding indicates that strawberry-tree fruit also likely has significant invertase activity allowing them to have a glucose/fructose ratio higher than 1, following other wild fruits, mainly wild red-bilberries, and blackthorn fruits [7,30].

Mineral rates proved a high variability among samples and high coefficients of variation, ranging from almost 14% for potassium to 73.9% for sodium. Looking at the level of individuals among the five surveyed regions, the ashes and mineral contents of fruits might be influenced by geographical location. As can be noticed, ashes content ranged from 1.9 to 2.8 g/100 g on a DW basis, which is higher than reported by Ruiz-Rodríguez et al. [10], for the same species (values ranged between 0.7 and 1 g/100 g). Phosphorus (P) was the mineral element the most abundant in mineral composition showing the highest contents (ranged between 55.5 and 86.9 mg/100 g), followed by potassium (K) (ranged between 53.4 and 61.1 mg/100 g), while sodium (Na) was presented in lowest amounts (ranged between 3.1 and 28.5 mg/100 g). Furthermore, regarding the trace elements contents, Iron (Fe) reached the highest value (ranged between 24.3–42.8 mg/100 g), while zinc (Zn) and manganese (Mn) exhibited slightly lowest amounts, with contents between 2.8–5.6 mg/100 g and 3.4–4.3 mg/100 g, respectively. In other work, fruits collected in Turkey and Croatia had K (119 mg/100 g, 118.61 mg/100 g); P (12.6 mg/100 g, 19.99 mg/100 g); Fe (1.25 mg/100 g, 1.29 mg/100 g); Zn (2.602 mg/100 g, 0.45 mg/100 g); and Mn (0.197 mg/100 g, <0.99 mg/100 g) [31,32].

The order of micronutrients making up the mineral composition was in agreement with the previous studies [10,16], but there are some differences in the contents of each element, especially for traces elements, where Moroccan fruits contain the highest levels of iron (Fe), zinc (Zn) and manganese (Mn), compared with values found in some Mediterranean fruit, [26,31,32].

The micronutrients (P, K, and Na) and the traces elements (Fe, Mn, and Mn) were the most variable parameter depending on the region of harvest, while ashes amount was almost stable over the surveyed samples collected from the different areas. It was reported that mineral composition is highly influenced by edaphic and environmental conditions such as precipitation and humidity [33,34]. These factors tend to largely influence mineral element contents since they can favor situations of physiological stress for the plant, which induce a defense process in which the minerals could act as co-factors to regulate the plant’s metabolic pathways [35].

According to RDA values reported in (Table 6), iron (Fe) was the highest contributor to mineral composition, the consumption of a 100 g portion of Moroccan fruit exceeds three times more the daily iron intake for men and two times more the daily iron intake for women (average iron intakes of 8 mg/d and 12.5 mg/d for men and women, respectively), followed by manganese which exceeds the daily intake (2.3 mg/d and 1.8 mg/d for men and women, respectively) by 70% for men and 94.4% for women, and finally the zinc with a daily intake of 36.1–49.6%, for men and women, respectively. While phosphorus was the lower contributor, with a portion intake of 9.4% for men and women, this means that Moroccan fruit provides a higher daily mineral intake than reported for Spanish fruit [10].

Regarding the main role of mineral in human health, especially the potassium for its function in the maintenance of several organs as heart, bone, muscle, and heart, in addition to its essential role in immune systems preservation [34], microelements such Fe, Zn, and Mn are known for their biological functions to maintain human health. This is mainly related to their potent antioxidant potential (protection of the body from the effect of redox reactions by scavenging free radicals). Moreover, zinc is a potent anticancer agent and has a main role at the cellular level in the human body [10,35].

## 5. Conclusions

Data on the nutritive and mineral composition of Moroccan *A. unedo* fruit were studied for the first time for this number of samples and traits. The present study has covered almost a range of Moroccan *A. unedo* distribution. The most abundant macronutrient was carbohydrates, total sugar, and dietary fiber. Moreover, fruits are very rich in phosphorus, potassium, and iron. The sugar composition was characterized by the abundance of glucose and fructose in all samples over surveyed regions whereas sucrose was recorded at very low levels.

The quantitative heterogeneity of wild Moroccan *A. unedo* showed the existence of high intrapopulation variability within the samples, particularly for the mineral content. The variability in nutritional composition observed among samples could be explained by genetic and environmental effects. However, further study on the genetic characterization using molecular markers is in progress in order to understand the pattern of variation in the nutritional value and the particular sugar profile of Moroccan strawberry tree genotypes, compared with the fruits grown in the other geographies of the Mediterranean basin, namely Portugal, Spain and Algeria

The contribution of *A. unedo* fruit to the total dietary intake that includes dietary fiber, protein, carbohydrates, and minerals was estimated for the first time in Morocco grown samples. The obtained results highlight the use of Moroccan strawberry-tree fruit as a healthy alternative for fruit production, especially for free-sugar composition, which is characterized by a ratio fructose/glucose lower than 1, which is consistent with health recommendations, in particular for people with diabetes. After proving the great nutritional quality of this wild fruit, it is necessary to provide further studies first, on the genetic variability of this species in Morocco and then on their phytochemical composition to demonstrate their therapeutic potential and to promote their potential utilization in the food industry.

## Figures and Tables

**Table 1:** Number of samples and altitude of the 12th locations prospected.

Regions	Locations	Number of Samples and Codes	Altitude (m)
OUL	HR	4 (HR1—HR4)	963
	AKH	3 (AKH1—AKH3)	989
	BS	4 (BS1—BS4)	1180
OUJ	Osy	3 (Osy1—Osy3)	1066
CH	Hc	7 (Hch1—Hch7)	698
BA	Ja	6 (Ja1—Ja6)	527
	Mya	3 (Mya1—Mya3)	444
	Dazia	4 (Dazia1—Dazia4)	487
	BH	5 (BH1—BH5)	721
SQ	KD	2 (KD1—KD2)	340
	Kh	2 (Kh1—Kh2)	300
	Sq	2 (Sq1—Sq2)	326

**Table 2:** Nutritive value average of *A. unedo* fruits harvested from Morocco.

Parameters	Number of Samples	Mean ± SE	Min	Max	CV (%)
Moisture (g/100 g DW)	45	9.87 ± 0.10	7.61	13.56	12.79
Fat (g/100 g DW)	45	3.44 ± 0.06	1.56	5.16	23.44
Ash (g/100 g DW)	45	2.24 ± 0.03	1.53	3.87	18.51
Protein (g/100 g DW)	45	2.83 ± 0.04	1.69	3.97	16.86
pH	45	3.94 ± 0.01	3.75	4.19	3.00
Total sugars (g EG/100 g DW)	45	60.95 ± 0.28	52.05	67.15	5.48
Dietary fiber (g/100 g DW)	45	16.07 ± 0.17	11.02	20.10	12.90
Carbohydrates (g/100 g)	45	81.59 ± 0.12	78.24	84.75	1.84
Energetic value (Kcal/100 g)	45	368.73 ± 0.65	346.48	384.11	2.07

**Table 3:** The average value of macro and microelements in the mineral composition of Moroccan *A. unedo* fruits.

	Parameters (mg/100 g DW)	Number of Samples	Mean ± SE	Min	Max	CV (%)
Macroelements	P	45	63.70 ± 1.19	37.56	96.57	21.74
	K	45	57.38 ± 0.70	39.00	89.00	14.24
	Na	45	15.09 ± 4.99	1.00	83.00	73.94
Microelements	Mn	45	3.85 ± 0.18	1.60	26.00	54.01
	Fe	45	28.74 ± 0.99	10.80	68.10	40.20
	Zn	45	3.94 ± 0.17	2.30	12.50	50.34

**Table 4:** Summary of the variation in nutritive value and mineral composition and the classification of harvested samples across groups generated by using Cluster analysis.

Variables	Group I (N = 3)				Group II (N = 10)				Group III (N = 14)				Group IV (N = 5)				Group V (N = 8)				Group VI (N = 4)				Ungrouped Sample
	Mean	SD	Min	Max	Mean	SD	Min	Max	Mean	SD	Min	Max	Mean	SD	Min	Max	Mean	SD	Min	Max	Mean	SD	Min	Max	−
Moisture	12.02	1.15	10.82	13.14	10.48	1.22	9.00	13.53	9.54	0.89	7.62	10.84	9.34	1.26	7.91	11.31	9.93	1.16	8.17	11.74	8.89	0.82	7.84	9.57	8.33
Protein	3.21	0.59	2.52	3.65	2.48	0.23	2.05	2.86	3.23	0.28	2.88	3.23	2.36	0.35	1.90	2.72	2.77	0.35	2.12	3.16	2.83	0.51	2.36	3.52	2.42
Dietary fiber	13.23	2.54	11.05	16.03	15.30	2.05	12.27	18.36	16.19	2.39	12.95	16.19	16.68	1.04	15.25	17.68	17.11	0.92	15.77	18.25	16.49	0.98	15.54	17.87	17.53
Fat	2.21	0.59	1.72	2.87	3.42	0.58	2.60	4.49	3.33	0.75	1.86	3.33	2.79	0.44	2.21	3.24	4.27	0.48	3.61	5.08	3.84	0.74	2.80	4.55	4.19
Total sugar	62.87	1.05	61.86	63.96	62.87	2.43	58.16	66.95	60.82	3.05	57.72	60.82	57.62	3.50	52.41	61.06	61.96	2.14	59.67	65.24	58.88	4.71	52.80	64.26	55.00
Ash	2.88	0.87	2.09	3.82	2.06	0.36	1.71	2.86	2.41	0.36	1.90	3.17	2.13	0.28	1.70	2.44	2.19	0.24	1.84	2.58	1.96	0.16	1.81	2.12	2.22
P	58.97	16.60	43.79	76.71	49.34	6.43	37.85	59.06	59.66	8.42	46.55	59.66	72.66	6.52	65.83	81.20	77.47	8.78	66.12	91.11	70.45	12.89	52.73	83.23	95.94
K	57.89	9.52	47	64.67	54.93	10.14	40.00	71.33	55.52	4.29	47.00	55.52	59.33	4.35	54.00	65.67	63.50	11.55	52.00	87.33	53.58	4.47	48.00	57.67	62.67
Na	17.22	12.40	3.67	28.00	10.60	11.05	1.67	30.00	21.38	13.10	1.33	21.38	15.20	11.61	4.67	29.33	3.17	1.46	1.33	5.67	68.00	9.89	60.00	81.33	3.33
Fe	19.53	11.01	11.03	31.97	28.32	13.50	11.87	50.03	27.90	8.55	14.57	27.90	27.07	6.81	15.10	31.47	30.03	12.45	13.97	54.43	29.37	3.36	24.83	32.93	67.80
Zn	3.04	0.49	2.63	3.6	3.88	1.64	2.40	7.30	3.67	0.97	3.00	3.67	4.96	3.53	2.73	11.13	3.54	0.67	2.50	4.43	3.18	1.01	2.60	4.70	12.30
Mn	3.15	1.39	2	4.7	3.38	0.99	1.67	4.70	3.88	0.62	2.73	3.88	3.71	0.48	3.17	4.23	4.51	2.43	2.97	10.47	4.18	1.06	2.97	5.53	4.37

**Table 5:** Total sugar content (gEG/100 g) and soluble sugars (g/100 g DW) of *A. unedo* L. fruits samples over surveyed regions.

Regions	OUL	OUJ	CH	BA	SQ
Total sugar	61.04 ± 0.25 ^b^	57.79 ± 0.19 ^c^	62.11 ± 0.54 ^a^	62.01 ± 0.43 ^a^	57.81 ± 1.13 ^c^
Glucose	14.87 ± 0.005 ^b^	14.84 ± 0.005 ^b^	12.19 ± 0.017 ^c^	15.17 ± 0.017 ^a^	11.57 ± 0.01 ^d^
Fructose	11.65 ± 0.01 ^b^	11.12 ± 0.01 ^b^	10.27 ± 0.008 ^c^	13.08 ± 0.01 ^a^	8.73 ± 0.003 ^d^
Sucrose	4.16 ± 0.02 ^d^	7.94 ± 0.01 ^b^	8.11 ± 0.005 ^a^	5.34 ± 0.01 ^c^	5.34 ± 0.01 ^c^

Data are expressed as means ± Standard Error (SE). All measurements are analyzed in triplicate (*n* = 3). The values in the same raw with different letters (a to d) differ significantly at *p* < 0.05.

**Table 6:** Contribution to Recommended Dietary Allowances (RDA) referred by Trumbo et al. [27], to the Moroccan *A. unedo* fruits (in dry weight), compared to the Spanish strawberry tree (in fresh weight).

Nutrients and Mineral Elements	Recommended Intake (mg/day) [27]	Contribution to RDA (%) in Moroccan Fruits	Contribution to RDA (%) in Spanish Fruits [10]
Carbohydrates	130,000 ^a^	62.76 ^a^	18.1 ^a^
	130,000 ^b^	62.76 ^b^	18.1 ^b^
Protein	56,000 ^a^	5.05 ^a^	1.6 ^a^
	46,000 ^b^	6.15 ^b^	1.9 ^b^
Dietary fiber	38,000 ^a^	42.28 ^a^	42.6 ^a^
	25,000 ^b^	64.28 ^b^	64.8 ^b^
Fe	8 ^a^	359.25 ^a^	11.1 ^a^
	18 ^b^	159.66 ^b^	4.5 ^b^
Mn	2.3 ^a^	167.39 ^a^	3.5 ^a^
	1.8 ^b^	213.8 ^b^	4.5 ^b^
Zn	11 ^a^	35.81 ^a^	4.2 ^a^
	8 ^b^	49.25 ^b^	5.8 ^b^
P	700 ^a^	9.1 ^a^	-
	700 ^b^	9.1 ^b^	

^a^ For males. ^b^ For females.

## Data Availability

Not applicable.
